# Intravenous Dexketoprofen versus Intravenous Paracetamol for Dysmenorrhea: A Randomized Controlled Trial

**DOI:** 10.4274/balkanmedj.2016.0536

**Published:** 2018-07-24

**Authors:** Mustafa Serinken, Cenker Eken, Özgür Karcıoğlu

**Affiliations:** 1 Department of Emergency Medicine, Pamukkale University School of Medicine, Denizli, Turkey; 2 Department of Emergency Medicine, Akdeniz University School of Medicine, Antalya, Turkey; 3 Clinic of Emergency Medicine, İstanbul Training and Research Hospital, İstanbul, Turkey

**Keywords:** Dexketoprofen, dysmenorrhea, intravenous, paracetamol, treatment

## Abstract

**Background:**

Dysmenorrhea is one of the most common acute pain disorders among women of reproductive age.

**Aims:**

To compare the effects of IV paracetamol with dexketoprofen in patients presenting with primary dysmenorrhea to the emergency department.

**Study Design:**

Randomized controlled trial.

**Methods:**

Patients over 18 years old presenting with pelvic pain related to menstruation were eligible for the study. Study patients received 1 g paracetamol or 50 mg dexketoprofen in 100 mL normal saline with a 4-5 minute infusion via the intravenous route. Pain intensity was measured by a visual analog scale at 15 and 30 minutes. Patients were randomized and assigned to either of two study arms via sealed envelopes. Study drugs were identical in color, and thus both personnel and patients were blinded to the study drug. The dexketoprofen group comprised 49 patients, and the paracetamol group had 50 patients in the final analysis.

**Results:**

The mean age of the study subjects was 20.9±2.5 and the mean duration of the pain was 1.9±1.7 (median: 1, interquartile range: 1 to 2) hours. Both dexketoprofen (median change: 33, 95% CI: 24 to 38) and paracetamol (median change: 21, 95% CI: 12 to 32) effectively reduced the pain at 15 minutes, which was repeated at 30 minutes (median change: 63, 95% CI: 57 to 65 vs 55.5, 95% CI: 50 to 59, respectively). Pain improvement in the dexketoprofen group was better than in the paracetamol group at 15 (median difference: 8, 95% CI: 0 to 16, p=0.048) and 30 (median difference: 6, 95% CI: 1 to 12, p=0.028) minutes, which was statistically significant but not clinically significant.

**Conclusion:**

Dexketotoprofen has a better visual analogue scale score that is not clinically relevant compared to paracetamol.

Dysmenorrhea is one of the most common acute pain disorders, affects about 40% to 70% of women of reproductive age, and is a frequent cause that leads to time lost from work or school besides interfering with daily living (1,2). Dysmenorrhea is not uncommon and may be severe in up to 20% of women when it may influence daily activities (3).

Nonsteroidal anti-inflammatory drugs (NSAIDs) are drugs most commonly used in the treatment of primary dysmenorrhea. The NSAIDs that are approved by the US Food and Drug Administration for treatment of dysmenorrhea are: diclofenac, ibuprofen, ketoprofen, meclofenamate, mefenamic acid, and naproxen. NSAIDs are used related to findings that prostaglandins have a role in the pathogenesis of dysmenorrhea. Aspirin may not be as effective as these NSAIDs, and paracetamol as adjunctive therapy may be helpful for alleviating mild symptoms (1,4).

Randomized controlled trials showed that NSAIDs are effective options for relieving primary dysmenorrhea. In a systematic review of 73 randomized trials, NSAIDs were significantly more effective than placebo (OR: 4.50, 95% CI: 3.85-5.27) or paracetamol (OR: 1.90, 95% CI: 1.05-3.44) (1). NSAIDs represent the mainstays of drug therapy. However, there is no randomized trial comparing the effectiveness of paracetamol and dexketoprofen as treatment options in patients presenting to the emergency department (ED) for pain relief because of primary dysmenorrhea.

Intravenous (IV) paracetamol has shown to be an effective treatment option in various painful conditions in the ED (5,6,7). The present study aimed to compare the effects of IV paracetamol with those of dexketoprofen in patients presenting with primary dysmenorrhea to the ED.

## MATERIALS AND METHODS

### Study setting and design

This prospective randomized, double-blind, controlled study was conducted in the ED of a tertiary care hospital with an annual census of 75.000 patients between December 15, 2014, and April 15, 2015. The central ethics committee approved the study (2014/70177). The clinicaltrial.gov ID is NCT02373514. The study was planned as a superiority trial with two intervention arms, IV dexketoprofen, and paracetamol. The funding of the study was provided by the authors.

### Selection of participants

Patients over 18 years old presenting with pelvic pain related to menstruation were eligible for the study. All consecutive patients who presented with dysmenorrhea as their chief complaint were enrolled in the study. The analyses focused on the severity of pain, rather than on the length of time it lasted. Patients with signs of peritoneal irritation, allergy to the study drugs, history of renal and liver failure, alcohol or drug abuse and those whose received painkillers within the last six hours, or refused to give informed consent were excluded from the study.

Patients were enrolled in the study 24 hours/7 days a week. A senior resident determined the eligibility of patients. Diagnosis of dysmenorrhea was made by pelvic pain related to menstruation and history of painful cycles that resembled the present one. A normal physical examination without a remarkable pathology was also accepted as an eligible diagnosis.

### Interventions

Study patients received 1 gr paracetamol (Perfalgan, Bristol Myers, Italy) or 50 mg dexketoprofen (Arveles, UFSA, Turkey) in 100 mL normal saline with a 4-5 minute infusion via the IV route. Study drugs were identical in color. Assignment of patients to one of the study arms was established in a 1:1 ratio according to eight computerized randomization blocks performed before the study by a person blinded to the study. Patients eligible for the study were assigned to one of the study arms by designated numbers kept in an opaque envelope. A study nurse prepared the study drug and another nurse unaware of the study drug administered it to the patient. Physician, the nurse administered the drug, and the patients were all blinded to the study drug.

### Methods of measurement

A 100 mm visual analog scale displaying the numbers as 10, 20…100 (Zero; no pain and 100 mm; the worst pain) was used to measure the intensity of the pain. Pain measurements of patients were carried out at baseline, 15, and 30 minutes after administration of the study drug. Patients were blinded to previous VAS scores. The need for rescue drug and adverse events were also recorded on the study form. Drug-related side effects attributed to either of the drugs were evaluated in detail. Nausea was not accepted as a side effect if it existed before administration of the drug.

### Outcome measures

The primary outcome measure was pain relief at 15 and 30 minutes after administration of the drug. Secondary outcome measures were the need for rescue drug and adverse effects secondary to study drugs.

### Statistical analysis

Study data were analyzed with MedCalc, Statistical Package for Social Sciences 18.0 (SPSS Inc.; Chicago, IL, USA), and Confidence Interval Analysis software. Numerical data were presented as mean ± standard deviation and median interquartile range (IQR) and frequency data as rates. Study data were also expressed with 95% confidence interval (95% CI). Normality analysis was performed by the Kolmogorov Smirnov test. Two-group comparisons for numerical data were performed by Mann-Whitney U test and chi-square test for frequency data.

Power analysis gave the recommendation of a sample of 37 patients in each group, for 95% power and a standard deviation of 19 mm. All hypotheses were constructed as two-tailed, and an alpha-critical value of 0.05 was accepted as significant. All analyses were performed according to the intention-to-treat principle.

## RESULTS

**Figure 1:**
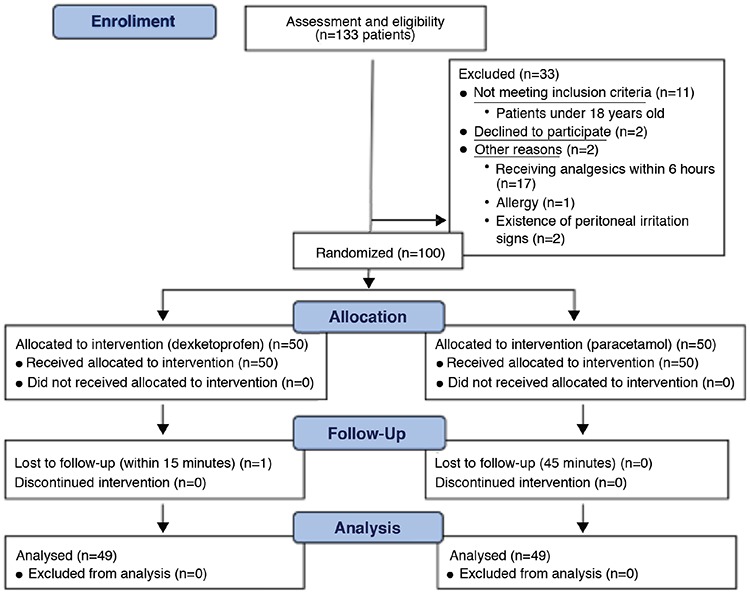
Patient flow chart.

A total of 133 patients were eligible for the study. Thirty-three patients were excluded from the study for various reasons, and one patient decided to withdraw from the study after 10 minutes of randomization and left the ED without rating the VAS scores (Figure 1). Forty-nine patients in the dexketoprofen group and 50 patients in the paracetamol group were included in the final analysis.

The mean age of study subjects was 20.9±2.5 years, and the mean duration of pain was 1.9±1.7 (median: 1, IQR: 1 to 2) hours. There was no difference between study groups regarding age and duration of pain (Table 1).

**Figure 2:**
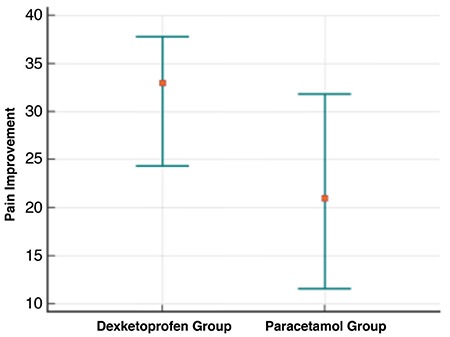
Pain improvement at 15 minutes in each study group.

**Figure 3:**
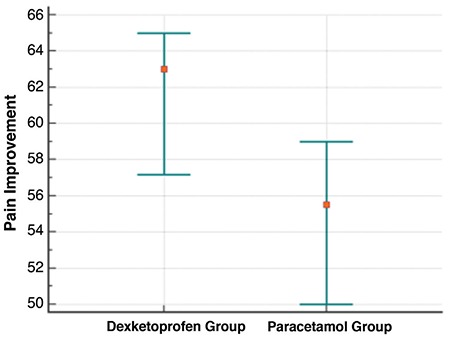
Pain improvement at 30 minutes in each study group.

Both dexketoprofen (median change: 33, 95% CI: 24 to 38) and paracetamol (median change: 21, 95% CI: 12 to 32) effectively reduced pain at 15 minutes (Figure 2, Table 2) and at 30 minutes (median change: 63, 95% CI: 57 to 65) vs 55.5, 95% CI: 50 to 59; respectively) (Table 2, Figure 3). Pain improvement in the dexketoprofen group was better than that in the paracetamol group at 15 (median difference: 8; 95% CI: 0 to 16) and 30 (median difference: 6; 95% CI: 1 to 12) minutes, which was statistically significant but not clinically significant (Table 3).

One patient (2%) in dexketoprofen group and 4 patients (8%) in paracetamol group needed rescue drug (p=0.37). One patient in the dexketoprofen group reported dry mouth, and one patient had nausea, and one patient had both nausea and vomiting in the paracetamol group.

## DISCUSSION

The present study showed that either IV dexketoprofen or IV paracetamol effectively provided pain relief in patients presenting with primary dysmenorrhea to the ED. Despite better VAS scores with dexketoprofen over paracetamol at both 15 and 30 minutes, this difference was not clinically significant. Dexketoprofen is also associated with a reduced need for rescue drug but did not have statistical significance.

Dysmenorrhea, which reflects itself as severe colicky abdominal pain, is attributed primarily to high levels of prostaglandins in the body. NSAIDs act by decreasing the synthesis of prostaglandins by blocking the cyclooxygenase (COX) enzyme. Dexketoprofen is a non-selective NSAID of the aryl-propionic acid group containing the active S-enantiomer of racemic ketoprofen. Dexketoprofen is an NSAID with a relatively short half-life and rapid onset of action (8).

Paracetamol provides its analgesic effect through central anti-nociceptive actions, specifically by inhibiting COX-3, which is a variant of COX-1. In addition, evidence suggests that paracetamol activates serotoninergic descending pathways that inhibit nociceptive signal transmission within the spinal cord. The most significant advantage of paracetamol is that it does not cause gastrointestinal bleeding or dyspepsia (9).

The current medical literature regarding dysmenorrhea consists mostly of studies examining oral forms of painkillers. We do not have sufficient data regarding the effects of parenteral painkillers in the ED. A Cochrane meta-analysis reported that NSAIDs achieve pain relief between 17% and 95% (mean, 67%) of women with an NNT (number of patients needed to treat) value of 2.1 compared with placebo for three to five days albeit gastrointestinal side effects (nausea, vomiting, and/or diarrhea) are still a concern (10). However, it is important to note that paracetamol remains the preferred choice for dysmenorrhea among young women around the world (11). For example, 57% of young women in Hong Kong use paracetamol for dysmenorrhea, which may be related to its safe side effect profile (1).

Despite its antipyretic and analgesic effects, paracetamol has little or no effect on inflammatory pathways. The analgesic effect of paracetamol partly emerges through supraspinal activation of descending serotonergic pathways, but its primary site of action may still be selective with variable inhibition of prostaglandin production (12). For acute pain management, IV paracetamol is both a safe and effective drug despite that its mechanism of action remains a controversial issue. Although most studies are interested in post-operative pain, IV paracetamol has been found to be as effective as opioids (5,6). IV paracetamol is thought to differ from both available IV opioids and NSAIDs by not being associated with adverse effects such as nausea, vomiting, and respiratory depression that are typically seen after opioid use, or the platelet dysfunction, gastritis, and renal toxicity that are related to NSAIDs. In the present study, the incidence of side effects was also rare, and this might be related to pain management.

A retrospective analysis of patients presenting with dysmenorrhea to the ED by Ayan et al. (13) reported that IV paracetamol had better pain relief than intramuscular diclofenac sodium at 30 minutes. However, the study is a retrospective chart analysis rather than a randomized controlled trial.

It is unclear whether specific NSAIDs are more effective or safer than others are. Although some studies have not reported differences in effectiveness, others have stated that fenamates (mefenamic acid, tolfenamic acid, flufenamic acid, meclofenamate, bromfenac) may have slightly better efficacy than phenylproprionic acid derivatives (ibuprofen, naproxen). Both fenamates and phenylproprionic acid derivatives inhibit prostaglandin synthesis, but fenamates also block prostaglandin action, which may be responsible for their enhanced effectiveness in some studies (1,14,15). A systematic review by Zhang and Li Wan Po (1) reported that ibuprofen had the most favorable risk-benefit ratio among other NSAIDs and aspirin. They reported that paracetamol was less effective than NSAIDs but pointed out that their investigation was only one study and additional studies need to be conducted.

There are some limitations to this study. This study is a superiority trial, and we cannot conclude that the two drugs are equal. One patient withdrew informed consent and left the ED without assigning pain intensity on the visual analog scale. There is no follow-up data of study patients as to whether their pain recurred or they attended another medical facility because of their dysmenorrhea.

In conclusion, IV dexketoprofen has better VAS scores that are not clinically significant in both groups.

## Figures and Tables

**Table 1:**
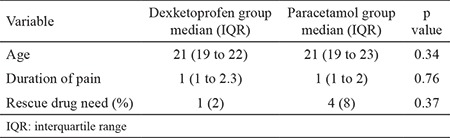
Comparison of demographics and rescue drug need in study groups

**Table 2:**
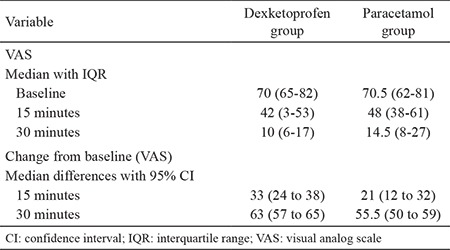
Change in pain intensity at 15 and 30 minutes for each study arm

**Table 3:**
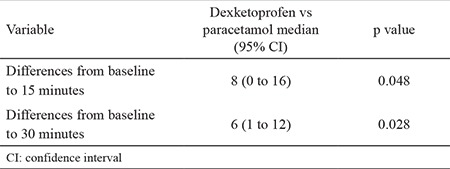
Comparison of pain improvements between the two groups
